# The Effect of Vitamin D Replacement on Patient with Subclinical Hypothyroidism: A Pilot Randomized Clinical Trial

**DOI:** 10.31661/gmj.v9i0.1592

**Published:** 2020-05-21

**Authors:** Babak Pezeshki, Ali Ahmadi, Aliasghar Karimi

**Affiliations:** ^1^Noncommunicable Disease Research Center, Fasa University of Medical Science, Fasa, Iran

**Keywords:** Vitamin D, Subclinical Hypothyroidism, Thyroid Stimulating Hormone

## Abstract

**Background:**

Subclinical hypothyroidism (SCH) is characterized by an elevated Thyroid Stimulating Hormone (TSH) with a normal T4. The prevalence of Vitamin D deficiency in patients SCH is high. Some studies suggested that Vitamin D supplements could be reduced serum concentration of TSH so improve SCH and prevent overt hypothyroidism. This study aims to explore the effect of vitamin D replacement on subclinical hypothyroidism.

**Materials and Methods:**

Fifty-nine patients, diagnosed with both subclinical hypothyroidism and Vitamin D deficiency by the Endocrinology outpatient clinics between January 2018 and March 2019, were included in this trial. The patients with overt hypothyroidism, cardiovascular risk factors, or positive TPO antibody, abnormal T4, and pregnant women were excluded from this study. The 40 subjects were investigated who received vitamin D supplements for two months. Analyses were conducted through paired-samples t-test and independent-samples t-test using SPSS 24 (Armonk, NY: IBM Corp).

**Results:**

The mean serum levels of TSH was decreased from 6.89 mIU/l in the pre-test to 3.34 mIU/l in the post-test, and the difference was found to be statistically significant at P<0.001.

**Conclusion:**

We found that the TSH mean level significantly dropped through the use of vitamin D supplements. Thus, it is recommended that all the patients with subclinical hypothyroidism be screened and treated with vitamin D supplements.

## Introduction

Subclinical hypothyroidism (SCH) is characterized by a high serum concentration of thyroid-stimulating hormone (TSH). Typically, serum concentrations of thyroid hormones are normal in SCH [1]. that is asymptomatic most of the time. However, it can present with symptoms of hypothyroidism [2]. The incidence of subclinical hypothyroidism has been reported in different studies to vary between 3-15% depending on the population studied [3-5]. However, there have been no studies conducted explicitly in the Iranian context to investigate the prevalence of SCH or to study its relation to vitamin D. Various studies have indicated that SCH can potentially be related to cardiovascular disease, congestive heart failure, and cognitive decline patients. Therefore, patients with SCH should be considered more seriously and checked for their risk of atherosclerotic cardiovascular disease and other risk factors [6-9]. Vitamin D has been found to play an essential role in maintaining an adequate serum level of calcium and phosphorus [10] and exerting an endocrine action on the cells of the immune system, generating anti-inflammatory and immunoregulatory effects [10-12]. However, the way vitamin D plays its role in autoimmunity is not yet completely recognized. Vitamin D deficiency has been seen in several autoimmune diseases, such as rheumatoid arthritis [13], systemic lupus erythematosus [14], type 1 diabetes mellitus [15], multiple sclerosis [16], inflammatory bowel diseases [17], autoimmune thyroid diseases (i.e., Hashimoto’s thyroiditis and Graves’ disease) [18-22]. So far, only two studies have been conducted on the relationship between subclinical hypothyroidism and vitamin D deficiency [23]. These studies have argued that treating vitamin D deficiency can reduce the TSH level. The current study is aimed at investigating the effect of vitamin D deficiency treatment in patients with subclinical hypothyroidism on reducing the TSH level.

## Materials and Methods

**Figure 1:**
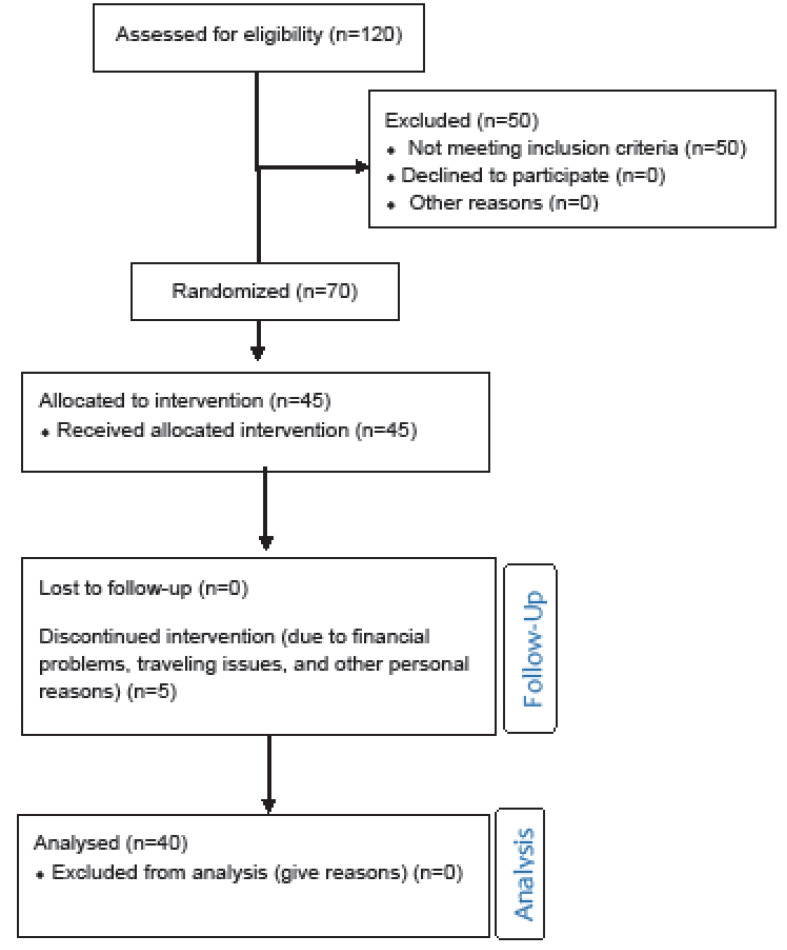
The flow chart of this study

The subjects were studied in the endocrinology outpatient clinic between January 2018 and March 2019. All the subjects were recruited at the endocrinology clinic of Fasa University of medical sciences. The protocol of this study was approved by the Institutional Review Board and Ethical committee of Medicine of Fasa university of medical sciences (code: IR.FUMS.REC.1397.115). This trial was registered in IRRCT.ir with code number: IRCT20190610043856N1. Seventy patients suffering from both SCH and Vitamin D deficiency were included in this clinical trial. The subjects were excluded from this study if they had one of these criteria: Consumption Vitamin D replacement in the last 15 months, overt hypothyroidism, taking medications that affect thyroid function such as steroid and L-thyroxine, TSH>10 mIU/l, have symptoms of hypothyroid, cardiovascular risk factors, positive TPO antibody, abnormal T4, and pregnant women. SCH was diagnosed by high serum TSH levels without symptoms of hypothyroid. Some of the subjects left the study, so the final sample included 40 subjects (5 males, 35 females). All of them received a vitamin D supplement (50000 IU) for two weeks as the standard treatment of vitamin D deficiency [15]. The consort flow chart of this study was shown in Figure-1. Vitamin D deficiency and insufficiency were defined as serum values of less than 30 ng/mL [24-28].

###  Blood Measurements

 A venous blood sample was collected in the Fasa University laboratory. Serum TSH and vitamin D were estimated using enzyme-linked fluorescence assay (ELFA). TSH levels between 0.25 -5 mIU/l were categorized as euthyroid, with levels higher than 10 mIU/l as overt hypothyroid, and with levels between 5-10 mIU/l as SCH, so the TSH upper normal limits for an adult is 4 mIU/l. Specific ELISAs measured 25 (OH)D. Vitamin D deficiency and insufficiency was considered if vitamin D levels were less than 30 ng/ml.

###  Statistical Analysis

 Analyses were conducted through paired-samples t-test and independent-samples t-test using SPSS software package (IBM SPSS Statistics, Version 24). Paired-samples t-test was employed to study the change in vitamin D and TSH values from pre-test to post-test. Finally, the correlation was used to see whether age was related to vitamin D absorption. The sample size was calculated by PASS Sample Size Software version 2019 (d=15, P=80, Confidence Level 95%).

## Results

**Figure 2:**
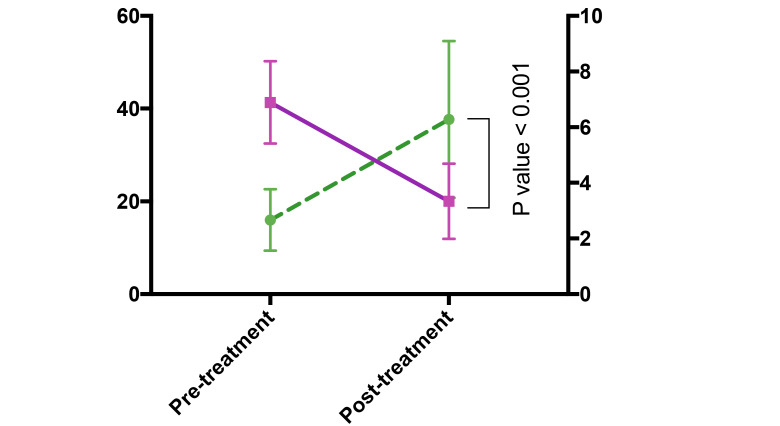
The relation between TSH and vitamin D during treatment

 The mean age was 36.7±13.5 years, and the mean TSH was 6.89 mIU/l. The serum level of vitamin D before treatment was 15.98 ng/mL. After Vitamin D replacement, the TSH value decreased to 3.34 mIU/l. This result indicated that the difference between the before and after treatment statistically significant (P-value<0.001). In other words, using vitamin D supplements has been effective in reducing TSH values. Figure-2 can depict this difference more clearly. To further explore the effect of vitamin D supplements on improving subclinical hypothyroidism, the mean vitamin D values have increased from 15.98 to 37.68 ng/ml. In comparison, the mean TSH values have decreased from 6.89 to 3.34 mIU/l, appearing in the normal TSH range. Table-1 indicates that these differences were statistically significant at (P-value<0.001), with large effect sizes of 0.78 and 0.62 for TSH and vitamin D, respectively, meaning that a significant increase in the vitamin D value has caused a significant decrease in the TSH value.

## Discussion

This clinical trial performed to investigate the efficacy of taking vitamin D supplements on improving SCH. Previous studies showed there is a link between serum concentrations of vitamin D and autoimmune thyroid diseases [20-23]. There is a negative correlation of vitamin D levels with serum concentrations of TSH in patients with SCH, so Vitamin D deficiency is significantly higher in patients with hypothyroidism versus the normal population [24,25]. As a consequence, vitamin D has a role in the pathogenesis to distribute the thyroid hormones [22]. In contrast, few studies indicated that hypothyroidism impaired the function of epidermal, so it distributes the synthesis of vitamin D on the skin. For that reason, vitamin D deficiency is common in these patients with hypothyroidism [26,27]. In patients with hypothyroidism, absorption, and activation of vitamin D might be impaired, so the replacement of vitamin D is necessary to improve the thyroid function. In consequence, they need to receive vitamin D supplements [28]. Several studies investigated the role of vitamin D supplements and thyroid hormones. Johani *et al*. [29] showed that the consumption of vitamin D supplements significantly reduced serum concentrations of TSH in patients with mild autoimmune SCH.

Mirhosseini, *et al*. [30] demonstrated that vitamin D supplement reduces the serum concentrations of TSH and prevents hypothyroidism in the future. They suggested the normal thyroid function needs adequate serum concentrations of vitamin D, so the daily intake of vitamin D supplements is beneficial and economical. The study suggests that vitamin D plays a role in subclinical hypothyroidism, although a causal relationship could not be established. In the current study, we found that giving vitamin D supplements can improve SCH by reducing serum concentrations of TSH, so patients who received vitamin D supplements for two months indicated a normal range of TSH, which concluded that treatment with vitamin D supplements has a significant impact on improving SCH by reducing TSH.

## Conclusion

 This small pilot study provided evidence for the role that vitamin D plays in improving SCH through the reduction of TSH, so the replacement of vitamin D may function as a protection against developing SCH to overt hypothyroid. This study suggests that SCH patients be regularly screened for their vitamin D deficiency and be treated by supplements. Also, age can play a role in the absorption of this vitamin, so it is recommended that older people receive vitamin D supplements.

 More clinical trials with larger samples are required to better depict the effect of vitamin D supplement to improving SCH and the protective effect of vitamin D against overt hypothyroidism in the future.

## Limitations of the Study

 Caution should be applied in generalizing the findings of this study as, like any other study, it may suffer from some limitations. The fact that the patients were selected through convenient sampling can limit the generalizability of the findings. Furthermore, the small number of subjects, non-blind, non-control, calls for more studies with larger samples.

## Conflict of Interest

 The authors declare that they have no competing interests.

**Table 1:** The Value of TSH and Vitamin D before and after Treatment

	**Pretreatment ** **(Mean ± Sd)**	**Post Treatment** **(Mean ± Sd)**	**Difference** ** (Mean ± Sd)**	**P-value **
**TSH mIU /l**	6.89 ± 1.48	3.34 ± 1.35	3.55 ±1.90	<0.001
**Vitamin D ng/ml**	15.98 ± 6.62	37.68 ± 16.92	21.70±17.28	<0.001
